# Emerging roles of noncoding micro RNAs and circular RNAs in bovine mastitis: Regulation, breeding, diagnosis, and therapy

**DOI:** 10.3389/fmicb.2022.1048142

**Published:** 2022-11-15

**Authors:** Weicheng Zong, Tianying Zhang, Bing Chen, Qinyue Lu, Xiang Cao, Kun Wang, Zhangping Yang, Zhi Chen, Yi Yang

**Affiliations:** ^1^College of Veterinary Medicine, Yangzhou University, Yangzhou, China; ^2^Joint International Research Laboratory of Agriculture and Agri-Product Safety, Ministry of Education, Yangzhou University, Yangzhou, China; ^3^Shaanxi Key Laboratory of Brain Disorders, Institute of Basic and Translational MedicineXi’an Medical University, Xi’an, China; ^4^Animal and Plant Inspection and Quarantine Technology Center, Shenzhen Customs, Shenzhen, China; ^5^College of Animal Science and Technology, Yangzhou University, Yangzhou, China

**Keywords:** bovine mastitis, micro RNAs, circular RNAs, biomarker, resistance breeding, therapy

## Abstract

Bovine mastitis is one of the most troublesome and costly problems in the modern dairy industry, which is not only difficult to monitor, but can also cause economic losses while having significant implications on public health. However, efficacious preventative methods and therapy are still lacking. Moreover, new drugs and therapeutic targets are in increasing demand due to antibiotic restrictions. In recent years, noncoding RNAs have gained popularity as a topic in pathological and genetic studies. Meanwhile, there is growing evidence that they play a role in regulating various biological processes and developing novel treatment platforms. In light of this, this review focuses on two types of noncoding RNAs, micro RNAs and circular RNAs, and summarizes their characterizations, relationships, potential applications as selection markers, diagnostic or treatment targets and potential applications in RNA-based therapy, in order to shed new light on further research.

## Introduction

Bovine mastitis, an inflammatory disease of the mammary gland, is recognized as one of the most troublesome and costly problems in modern dairy industry. There are several factors contributing to mastitis that is notorious for its high morbidity, including pathogenetic microbial infections (Nash et al., 2003), physical trauma and environmental hazards (Chen et al., 2021, 2022). Hitherto over 130 pathogens are demonstrated to be related to mastitis (Bradley, 2002). Over 80% of global economic losses (~35 billion USD) is attributed to reduced dairy production and inferior milk quality (especially in China), and monthly losses can be up to 76,000 USD per farm (He et al., 2020). More importantly, mastitis can pose a public health risk. Due to the abuse of antimicrobial agents in therapy and prophylaxis for food animals previously (Aarestrup, 2005), antimicrobial-resistant (AMR) pathogens can be frequently isolated from raw milk and animal feces (Holko et al., 2019; Rovira et al., 2019). However, except for the use of antibiotics, there is still no effective therapy that can be applied to the treatment of bovine mastitis on a large scale.

Immunization and the improvement of management concepts have traditionally been considered as potent approaches for preventing bovine mastitis (Ruegg, 2017). At the same time, alternative therapies are under investigation to replace the use of antibiotics in mastitis control, owing to the abovementioned AMR problem (El-Sayed and Kamel, 2021). Moreover, current vaccines fail to prevent mastitis for various reasons, such as nutrient level (Jing et al., 2021), mammary gland features, and the lack of suitable adjuvants or delivery systems (Rainard et al., 2022b). In general, treatment failure of mastitis is associated with delayed diagnosis, because most of diseased animals are suffering from chronic courses, also known as subclinical mastitis, making monitoring and controlling them more challenging (Ruegg, 2017; Cobirka et al., 2020). So far, somatic cells counting (SCC), the global standard for milk quality, has been used as an index for mastitis management (Ruegg, 2017), but SCC-value only indicates the pathological state, while the pathogenesis and associated details still need further laboratory diagnosis (Sanchez-Visedo et al., 2020). It is therefore important to identify new biomarkers for diagnosis of subclinical cases, as well as for our understanding of pathology of mastitis.

Disease resistance breeding is one of new strategies for sustainable dairy industry (Bronzo et al., 2020; Rasheed et al., 2020). In the past, it has mainly been genetic mutations in DNA, such as single-nucleotide polymorphisms (SNPs; Hou et al., 2012; Wang et al., 2014; Ju et al., 2018c), insertions and deletions (indels; Hu et al., 2022), or copy number variations (CNV; Molotsi, 2020), that served as a basis for predicting and demonstrating susceptibility of animals to different diseases. Recently, it has become increasingly apparent that endogenous RNA molecules from noncoding regions of genome play important roles in regulating biological process like inflammation and immunity (Do et al., 2021; Della Bella et al., 2022). Among them, the most well-known group is microRNAs (miRNA). With the increasing involvement of newfound noncoding RNA molecules, such as long noncoding RNAs (lncRNA) or circular RNAs (circRNA), a competitive endogenous RNA (ceRNA) network has been proposed (Qu et al., 2015), offering a new perspective for further transcriptome research. In addition, with the discovery of refinement and complex regulation processes involved in RNA molecules, comprehensive studies of ceRNA network could contribute to our knowledge of these unclarified biological processes to find novel therapeutic targets. Here, we review previous reports on the regulation of two noncoding RNA molecules (miRNAs and circRNAs) and summarize their potential in breeding and clinical applications as selection and/or diagnostic biomarkers in mastitis therapy.

## Micro RNAs

As a class of noncoding RNAs, micro RNAs, whose length is about 22 nucleotides, widely exist in eukaryote genome (Ha and Kim, 2014). So far, more than 38,000 precursor miRNAs, which express almost 49,000 mature miRNAs, have been discovered in 271 species (Kozomara et al., 2019). As early as 30 years ago, the first reported miRNA Lin-4 was discovered in *Caenorhabditis elegans* (Horvitz and Sulston, 1980; Lee et al., 1993), and in 2000, another miRNA Let-7 was found, thus initiating the study of miRNAs (Reinhart et al., 2000; Lagos-Quintana et al., 2001). Since then, numerous studies have been conducted on miRNAs, uncovering their roles in various biological processes, ranging from developmental regulation to disease progression (Treiber et al., 2019; Lu Q. et al., 2021; Della Bella et al., 2022).

### The biogenesis and regulatory mechanism of miRNA

**Figure 1:**
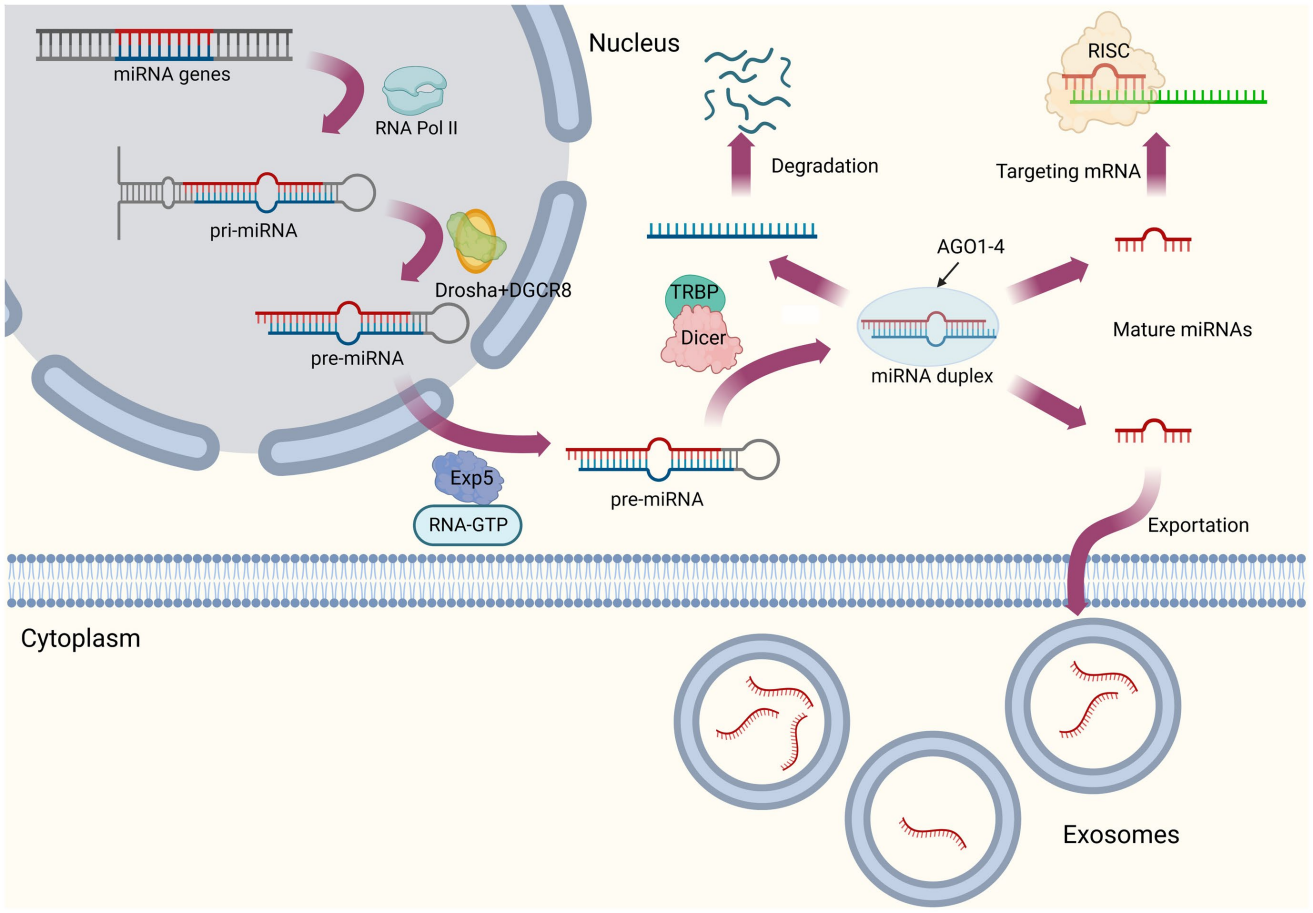
The biogenesis of miRNA.

Converting the primary miRNA transcript (pri-miRNAs) into mature miRNA requires several biogenesis steps. Canonical miRNA biogenesis pathway can be summarized as follows (Figure 1). First, pri-miRNAs are transcribed from introns of protein coding genes by RNA polymerase II (Pol II; Lee et al., 2004; Carthew and Sontheimer, 2009). The next step is to process pri-miRNAs into single hairpins termed precursor miRNAs (pre-miRNAs) by a nuclear protein complex, microprocessor (Gregory et al., 2004), which mainly consists of RNase III enzyme Drosha, double-stranded RNA (dsRNA)-binding protein (dsRBP), DiGeorge critical region 8 (DGCR8), and several auxiliary factors (Partin et al., 2020). They are subsequently exported to the cytoplasm *via* export receptor exportin 5 (Exp5) and processed into dsRNAs with a length of 20–25 nucleotides by RNase III-type enzyme Dicer (Ketting et al., 2001; Bohnsack et al., 2004). Finally, under the effect of the Argonaute (AGO) protein family, one strand of dsRNA is selected as mature miRNA (also known as guide strand), while the other strand (passenger strand or miRNA*) is discarded in a process termed as RNA-induced silencing complex (RISC) loading (Wu et al., 2020). Treiber et al. (2019) recently reviewed this part of non-canonical microRNA biogenesis pathway, which are often independent of one or several steps described before.

When mature miRNAs are exported to the cytoplasm, these molecules perform their functions at the occurrence of signals or targets. The regulatory mechanism of miRNAs is based on their characteristic RNA-induced silencing complex (RISC), while the degrees of complementary pairing of miRNAs with target mRNA sequences can be either complete or incomplete (Carthew and Sontheimer, 2009). In the former one, target mRNAs will be cut and degraded directly, while in the later form, miRNAs tend to act as translational inhibitors (Gaken et al., 2012). In general, miRNAs are less complementary to target mRNAs in animals, and their mode of action is primarily to inhibit the translation of target mRNAs (Lu Q. et al., 2021). Given that relationship between miRNAs and target mRNAs is not a simply one-to-one pattern, this means one miRNA could regulate multiple mRNAs, while one mRNA could be regulated by several miRNAs, indicating their interlacing interaction mechanism.

### Potential roles of miRNAs in bovine mastitis

**Figure 2:**
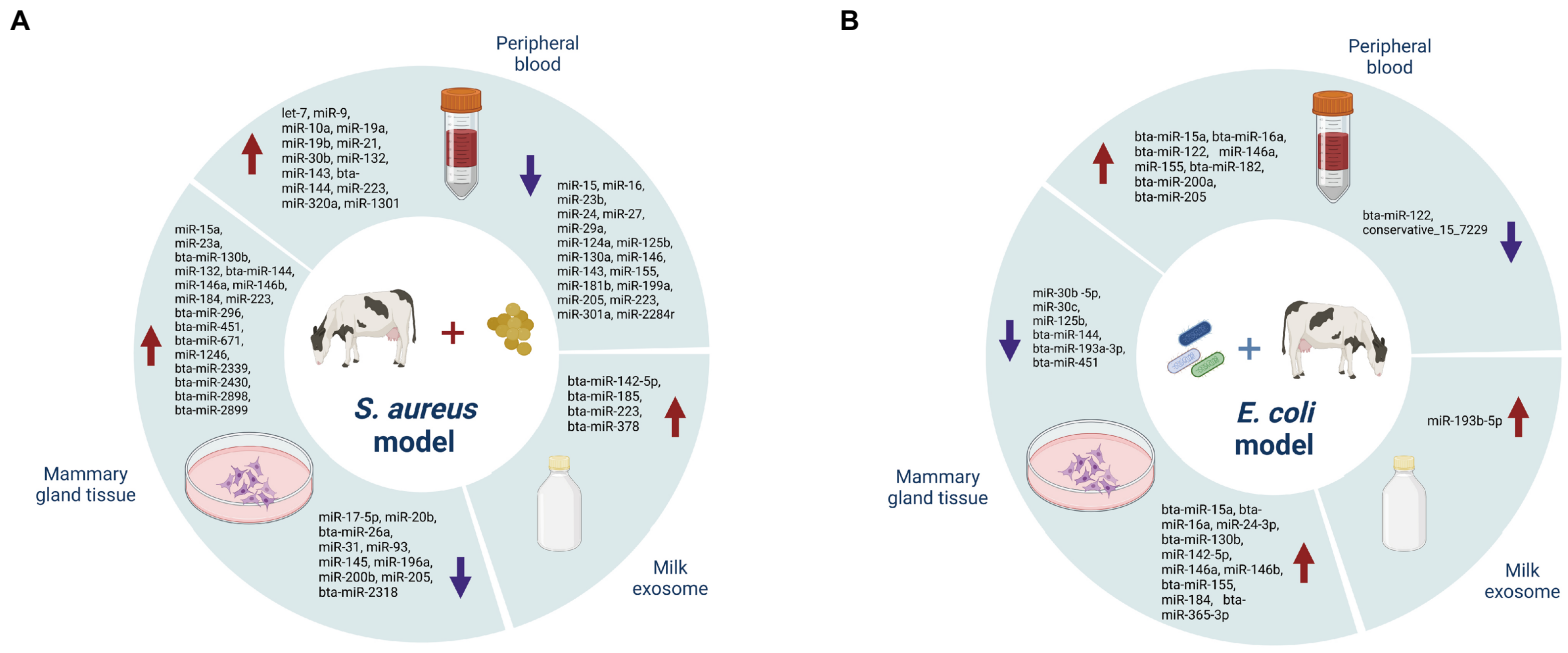
Differently expressed miRNAs in *Staphylococcus aureus* **(A)**, and *Escherichia coli* **(B)**, infection models.

In clinical cases, mastitis/mammary gland inflammation is caused by a pathogenic infection. Such pathogens can be classified into contagious and environmental types, which are represented by *Staphylococcus aureus* or *Streptococcus agalactiae*, and *Escherichia coli* or *Streptococcus uberis*, respectively (Cheng and Han, 2020). Usually, subclinical mastitis is caused by contagious factors characterized by chronic symptoms, while clinical mastitis often attributes to environmental factors and is characterized by acute symptoms. In practice, however, factors inducing mastitis are more complex for the high occurrence of cross infection (Luoreng et al., 2018a). Among pathogen-induced miRNA-mastitis model, *S. aureus* (Figure 2A) and *E. coli* (Figure 2B) have been well-studied, which are applied in simulating subclinical and clinical mastitis, respectively. Several sources of miRNAs exist in mastitis cows, including mammary gland tissue, peripheral blood cells and milk exosome (Table 1). The purpose of this section is to discuss identified miRNAs from these sources and their functions in regulating mastitis as well as potential therapeutic applications.

**Table 1:** Emerging mastitis related miRNAs from three different sources.

miRNAs	Sources	References
MiR-23a	Mammary gland tissues	Cai et al. (2021)
MiR-145		Chen et al. (2019a)
MiR-15a		Chen et al. (2019b)
Bta-mir-223 and bta-mir-21-3p		Fang et al. (2016)
Bta-miR-130b		Han (2019)
Bta-miR-2318, bta-miR-1777a, bta-miR-296, miR-2430, and miR-671		Hou et al. (2012)
Bta-miR-2899		Jiang et al. (2019)
Bta-miR-184, miR-24-3p, miR-148, miR-486, let-7a-5p, bta-miR-2339, miR-499, miR-23a and miR-99b		Jin et al. (2014)
Bta-miR-2426		Ju et al. (2018c)
Bta-miR-15a		Ju et al. (2018a)
Bta-miR-26a		Ju et al. (2018b)
Bta-let-7i, bta-miR-3596, bta-miR-21, bta-miR-27a, bta-miR-27b, bta-miR-151, bta-miR-184, bta-miR-200a, bta-miR-200b, bta-miR-205, bta-miR-29b-2 and bta-miR-130a		Lawless et al. (2013)
MiR-17-5p, miR-20b and miR-93		Li et al. (2013)
MiR-223, miR-132 and miR-1246		Li et al. (2015)
Bta-miR-144, bta-miR-451 and bta-miR-7863		Luoreng et al. (2018a)
MiR-125b		Luoreng et al. (2021a)
MiR-181a, miR-16 miR-31, and miR-223		Naeem et al. (2012)
Bta-let-7a-5p bta-let-7b, bta-let-7c, bta-let-7d, bta-let-7e, bta-let-7f, bta-let-7 g, bta-let-7i, bta-miR-1, bta-miR-100, bta-miR-101, bta-miR-103, bta-miR-106a, bta-miR-106b, bta-miR-107 and bta-miR-10a		Ozdemir (2020)
Bta-miR-193a, bta-miR-363, bta-miR-148b, bta-miR-205 and bta-let-7e		Passe Pereira et al. (2021)
MiR-223		Pu et al. (2017)
Bta-miR-24-3p, bta-miR-328, bta-miR-223, bta-miR-185, bta-mir-149-5p and bta-miR-874		Tucker et al. (2021)
Bta-miR-2898		Wang et al. (2014)
MiR-146a and miR-146b		Wang et al. (2016)
MiR-16 and miR-223	Peripheral blood cells	Chen et al. (2014)
MiR-9, miR-125 b, miR-155, miR-146 a and miR-223		Dilda et al. (2012)
MiR-21		Lai et al. (2021)
Let-7e, miR-150, miR-146b, bta-miR-200c, bta-miR-210, and bta-miR-193a		Lawless et al. (2014)
Bta-miR-223		Li et al. (2012)
Bta-miR-486, bta-miR-451, bta-miR-191, bta-miR-339b, mml-miR-486-5p, Bta-miR-25, bta-miR-342 and bta-miR-30e-5p		Li Z. et al. (2014)
MiR-200a, bta-miR-205, bta-miR-122, bta-miR-182 and conservative_15_7229		Luoreng et al. (2018b)
MiR-320a, miR-19a, miR-19b, miR-143, miR-205, miR-24, miR-1301 and miR-2284r		Luoreng et al. (2021b)
MiR-223 and miR-142-5p	Milk exosomes	Cai et al. (2018)
MiR-221		Cai et al. (2020)
MiR-21, miR-146a, miR-155, miR-222, and miR-383		Lai et al. (2017)
MiR-21, miR-146a, and miR-155		Lai et al. (2020)
Bta-miR-378 and bta-miR-185		Ma et al. (2019)
Bta-miR-223-3p		Saenz-de-Juano et al. (2022)
Bta-miR-142-5p and bta-miR-223		Sun et al. (2015)
MiR-193b-5p		Xu et al. (2021)

#### miRNAs as molecular markers in bovine mastitis resistance

SNPs within pre-miRNA regions may be responsible for a number of reported associations between SNPs, miRNAs, phenotypes and diseases (Wang Y. et al., 2021), which caught the eye of animal breeders in overcoming mastitis issues. Previous studies have been primarily focused on understanding the influence of genetic variations in the 3′ UTR (untranslated region) on the joint efficiency of differently expressed miRNAs. For example, Li et al. (2012) reported that SNP in the 3′ UTR of the bovine high-mobility group box protein 1 (*HMGB1*) gene could affect its target miRNA bta-miR-223, which is found to be upregulated in mammary gland tissues of mastitis. Similarly, bta-miR-2318, a downregulated miRNA in mastitis cows, is also repressed by SNP of *BOLA-DQA2*, which belongs to the bovine leukocyte antigen (*BOLA*) class II genes and participates in bovine immune response. Alpha-2-macroglobulin (A2M) has been reported to bind host or foreign peptides and particles, thereby serving as a defensive barrier in mastitis (Wang et al., 2012). Wang et al. (2014) further investigated the relationship between SNP in its exon 29 and target binding miRNA bta-miR-2898, indicating that bta-miR-2898 could regulate inflammation and immunity. However, these studies only demonstrated the binding efficiency repression of target miRNAs and predicted potential roles in mastitis, but further investigations as selection markers are still pending. It may be due to the lack of effective complementation strategies at RNA level in further experiment, and some progress has been made on this issue. Recently, various point mutation techniques for single bases in RNA editing have been proposed (Cox et al., 2017; Liu et al., 2020), some of which are applied to experimental treatment of genetic diseases caused by SNPs (Liao et al., 2020). In brief, as a result of the progress of this technology, we will be able to rediscover previous research findings, thereby gaining a better understanding of emerging variants associated with mastitis.

These years, as sequencing and bioinformatic technology have advanced (Stark et al., 2019), some new models based on miRNA and related mRNA or co-work protein also have been proposed and deepened our research on mastitis. By combining next-generation sequencing with advanced network biology methods and fluorescence activated cell sorting (FACS), Lawless et al. (2014) profiled the mRNA and miRNA expression in milk and blood CD14^+^ monocytes from animals infected *in vivo* with *S. uberis* at multiple time points. Meanwhile, they identified two miRNAs-Translation factor (TF) pairs, miR-233-STAT3 and miR-150-MyD88, both involved in Toll-like receptor (TLR), NOD-like receptor (NLR) and Retinoic acid-inducible gene I (RIG-I) signaling pathways. Notably, a number of metabolic processes were widely repressed during *S. uberis* infection, indicating that metabolic targets may play an important role during infection or immunological regulation (Palsson-McDermott and O’Neill, 2020). Moreover, Fang et al. (2017) proposed a model integrating sequence-based genome-wide association studies (GWAS) and RNA-Seq in studying genetic basis of mastitis, and further discussed the correlation between miRNA-network and GWAS markers (Fang et al., 2018). As a result, the new model improved the accuracy and reliability of previous prediction, indicating the contribution of microRNAs and their target networks to genetic variations in complex traits. Recently, a comprehensive approach based on the integrative analysis of miRNA and mRNA expression profiles was launched by Wang X. et al. (2021) and the regulatory functions of miRNAs, such as miR-23b-3p, miR-331-5p and miR-664 were emphasized again. A single association model analysis may only reflect part of the problem, but when multiple signals point to the same molecule, it is usually considered to be decisive. Thus, we recommend to use more analysis network crossing to identify, more reliable selection markers which is conductive to preventing mastitis in dairy breeding.

#### miRNAs as diagnostic biomarkers of bovine mastitis

California Mastitis Test (CMT) has been traditionally regarded as a cheap and easy method for detecting mastitis. Later, due to the gradual deepening of the pathogenesis of mastitis, some diagnostic technique based on etiology and immunology have been proposed, such as agglutination test (Zschock et al., 2005) and ELISA (Kano et al., 2016), which are widely used in dairy production practice. These years, as qRT-PCR methods and chemical probes based on nanomaterials continue to progress (Li J. et al., 2014), the detection of nucleic acid molecular markers in the peripheral blood or secretions to monitor disease progression has become a new direction in diagnostic technique. Although analysis based on mastitis related miRNAs lack further validation, these differently expressed endogenous molecules exhibit the potential to be regarded as biomarkers of diverse pathological patterns for diagnosis or as indicators of disease duration. As reported by Jin et al. (2014), for instance, five differentially expressed miRNAs (bta-miR-184, miR-24-3p, miR-148, miR-486 and let-7a-5p) have been identified in *E. coli* infected bovine mammary epithelial cells, while another four types (bta-miR-2339, miR-499, miR-23a and miR-99b) are unique to *S. aureus* infection. Additionally, miRNA expression also varies at different infectious timepoints, indicating a dynamic change over time. Compared with CMT, a classical mastitis assay based on somatic cells in milk (Ruegg, 2017), miRNAs in milk exosome are emerging as new biomarkers for more sophisticated diagnosis, as they are not only useful for reflecting whether cows are infected, but also help us understand the etiology and the progress of the disease (Colitti et al., 2020; Tzelos et al., 2022). To date, a batch of exosomal miRNAs have been screened in cows’ raw milk, such as miR-223 and miR-142-5p (Sun et al., 2015; Cai et al., 2018), and served as potential candidates for early detection of bacterial infection of the mammary gland. As research continues, miRNA biomarkers will benefit the high-accuracy early diagnosis of mastitis, especially in subclinical cases.

#### miRNAs as therapeutic targets of bovine mastitis

Traditionally, antibiotics have been considered as the priority choice for bovine mastitis treatment. However, exacerbated problems, such as antimicrobial resistance and antibiotic residue in animal products, give rise to the impact of antibiotic abuse on public health (Liu et al., 2016), leading to restrictions on uncontrolled antibiotic therapy in the dairy industry worldwide (El-Sayed and Kamel, 2021). Today, a sharp demand appears for new drugs and therapeutics, and miRNAs are identified as immuno-oncological targets (Calin et al., 2002), which also attracts the attention of veterinarians or veterinary researchers. The early research on mastitis aimed toward eradicating the infection by adjusting the abnormal levels of miRNA and inflammatory factors through traditional herbal medicine and animal nutrition. Quercetin, a secondary metabolite found in plants, has been reported to suppress the expression of miR-24-2, miR-146a and miR-181c, and decrease pro-inflammatory genes like *IL1B, IL6, CXCL8, TLR4* and *TNF* (Chuammitri et al., 2017). Apart from that, selenium is an important nutrient that has shown promise in regulating immune responses in the form of over 30 selenoproteins (Jing et al., 2021), reducing phosphorylation levels of NF-κB and MAPKs by downregulating miR-155 and upregulating miR-146a, thereby relieving mastitis (Sun et al., 2017; Zhang et al., 2019).

Disorders of miRNAs play an important role in diseases, especially in the pathological changes of cancer cells (Rupaimoole et al., 2016). As it has been proven that RNA interference (RNAi) is an effective strategy in *in vitro* experiments (Liu et al., 2017), correcting these disordered miRNAs is believed to have potential in disease treatment. Exogenous RNA molecules have therefore been considered as a promising strategy for molecular targeted therapy to adjust dysregulated miRNA transcription. Nevertheless, since RNA is unstable and sensitive to ribonuclease (Uslu and Wassenegger, 2020), it was thought unfeasible to apply exogenous RNA to regulate miRNA homeostasis *in vivo* and achieve similar therapeutic efficacy. Interestingly, in some past studies, complete exogenous miRNAs have been successfully isolated from food (Chen et al., 2010) and tissues (Zhang et al., 2012), and protective mechanisms such as exosome have also been discovered, confirming preceding assumptions.

Benefiting from previous efforts, miRNA-based treatments for many diseases are under rapid clinical development with the advantages of multiple targets, and there are serval successful practices in treating various kinds of human diseases (Rupaimoole and Slack, 2017), for example the locked nucleic acid (LNA) antimiRs of miR-221/222 were reported to remiss hepatic lipid accumulation, inflammation and collagen deposition in nonalcoholic steatohepatitis (Jiang et al., 2018), and the silencing of miR-122 caused by its LNA was proven effective during hepatitis C virus (HCV) infection and related liver diseases (Elmen et al., 2008). Meanwhile, multiple miRNA targets pertaining to one biological process and their varying expression levels in specific tissues bring about challenges in the identification of the most effective therapeutic candidates and strategies to safely deliver small molecules to selective tissues. Notably, circRNA, another newly found stable RNA molecule that will be discussed in the following chapter, is shown to extensively participate in post-transcriptional regulation as miRNA sponge *via* complementary sequences, which provides a novel reference for miRNA-targeted interference strategy. Moreover, it is confirmed that CRISPR/Cas9 nuclease system is able to knock out multiple miRNAs (Narayanan et al., 2016), which not only broadens the genetics approach, but also provides a powerful complement to gene therapy. Recently, new delivery systems such as engineering exosome (Choi et al., 2021) or selective organ targeting lipid nanoparticle (SORT-LNP; Algharib et al., 2020; Dilliard et al., 2021) are getting more and more mature, and miRNA-targeted gene therapy is expected to be a new breakthrough in bovine mastitis. Unlike human medical practice, veterinary medicine is often concerned with the balance between treatment cost and economic feedback. Despite the potential of miRNA-targeted gene therapy, there are several factors that limit the feasibility of this application to animal clinical practice, especially the high cost. Admittedly, the work of screening potential miRNA therapeutic targets has just started (Table 2), which provide buffer for technology decentralization with cost reduction. Therefore, we still have expectations that this strategy can be used in the treatment of bovine mastitis in the future.

**Table 2:** Corroborating studies of miRNA in regulating mastitis.

MiRNA	Model	Cell type	Target mRNA	Function	References
miR-214	Not mention	BMEC	NFATc3 TRAF3	Reducing inflammation by inhibiting MAP3K14, TBK1 and IL-6, IL-1β	Song et al. (2017)
miR-145	*S. aureus*	MAC-T	FSCN1	Promoting cell proliferation and facilitating the restoration of the damaged tissue	Chen et al. (2019a)
miR-221	Lipoteichoic acid (LTA)	RAW264.7	SOCS1	Upregulating JAK/STAT signaling in M1 macrophage polarization	Cai et al. (2020)
miR-23a	LTA	MAC-T	PI3K	Inhibiting inflammatory signaling pathway TLR2/MyD88/PI3K/AKT	Cai et al. (2021)
miR-125b	Lipopolysaccharide (LPS)	HEK 293T	NKIRAS2	Promoting inflammation by activating NF-κB and IL-6, TNF-α	Luoreng et al. (2021a)
miR-142-5p	LPS	MAC-T	BAG5	Promoting cell proliferation and facilitating the restoration of the damaged tissue	Lu J. et al. (2021a)

## Circular RNAs

Circular RNAs (circRNAs) are a group of single-stranded circular RNA molecules, which have been found to be abundantly expressed in eukaryotic cells (Dixon et al., 2005) since its initial discovery in viroid or viral genome (Sanger et al., 1976), and further widely reported in 1990s (Capel et al., 1993; Cocquerelle et al., 1993). Unlike other liner endogenous noncoding RNAs, circRNAs are generated *via* a non-canonical splicing manner described as “back-splicing” in which a downstream 5′ donor is covalently connected to an upstream 3′ accepter to form a closed-loop structure. This structure lacks the 3′ polyadenylated tail (Liang and Wilusz, 2014), deeming it undetectable *via* classical sequencing methods (Jeck and Sharpless, 2014). Additionally, it is more resistant to exonuclease-mediated degradation than liner transcriptions, such as Ribonuclease R (RNase R; Suzuki et al., 2006), suggesting of its potential in RNA drug delivery techniques (Qu et al., 2022). Additionally, circRNAs are found to be highly expressed in specific tissues and stages throughout animal genome, offering a potential biomarker or treatment target for disease detection (Wu et al., 2022). Moreover, considering its unique features, modified investigation methods have been proposed for further bioinformatical analysis and more functions have been predicted and verified during the interaction between pathogen and host, thus improving the insight of animal breeders (Wang J. P. et al., 2022; Xu et al., 2022). Research on animal circRNAs has become a hotspot in genetics and molecular biology with advances in techniques (Jeck and Sharpless, 2014).

### The regulatory mechanisms of circRNAs

**Figure 3:**
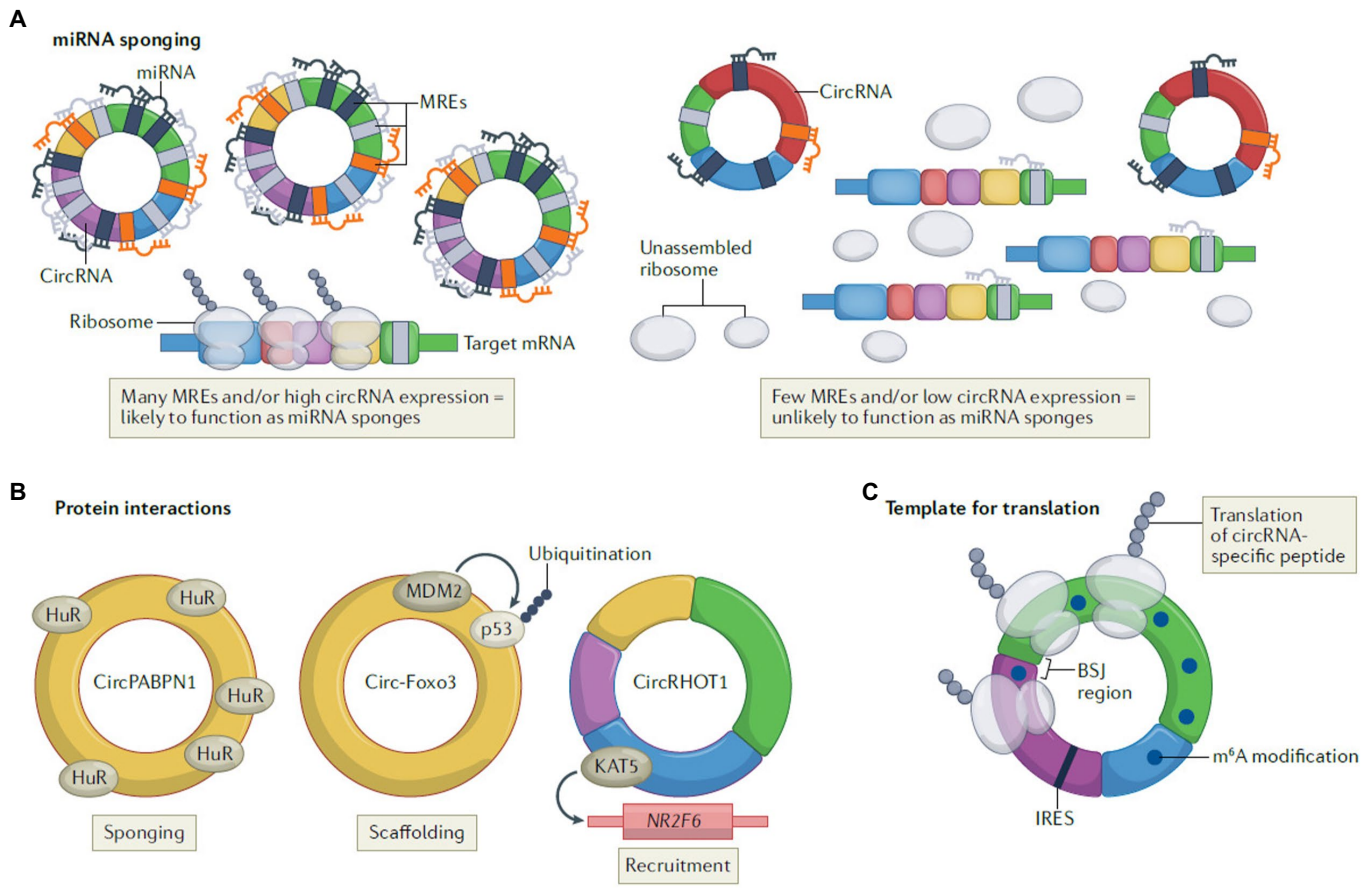
The regulatory mechanisms of circRNAs, including miRNA sponging **(A)**, protein interactions **(B)**, and template for translation **(C)** (Lasse S. Kristensen, Theresa Jakobsen, Henrik Hager, Jørgen Kjems; The emerging roles of circRNAs in cancer and oncology; Nature Reviews Clinical Oncology; 2021 [Springer Nature]).

In addition to be abundantly expressed in eukaryotes, circRNAs have various biological functions with complex and diverse action mechanisms. According to Kristensen et al., functions of circRNAs are classified into three regulatory mechanisms (Kristensen et al., 2022; Figure 3).

#### miRNA sponging

The function of circRNAs as miRNA sponge depends on their complementary sequences to target miRNAs, which is also known as miRNA reaction elements (MRE). By taking up target miRNAs, specific miRNAs can be prevented from interacting with or repressing target mRNA.

Take the most famous sponge ciRS-7 as an example, its target miR-7 contains over 60 binding sites and is proved to function as ceRNAs in certain tissues (Hansen et al., 2013). The lack of enough MREs, however, prevents all circRNAs from binding with target miRNAs as expected (Guo et al., 2014).

#### Protein interactions

In addition to miRNAs, there are also interactions between RNA binding protein (RBP) and circRNAs. Specifically, circRNAs function as protein sponges or inhibitors by acting as scaffolds to bring different proteins into proximity or recruiting them to specific subcellular compartments. In breast cancer cells, Circular RNA Forkhead box O3 (circ-Foxo3) has been demonstrated to facilitate ubiquitination and degradation of mutant p53 tumor suppressor protein (p53) by binding p53 and the E3 ubiquitin ligase mouse double minute 2 (MDM2) as the tumor suppressor. Meanwhile, it also prevents degradation of the pro-apoptotic transcription factor forkhead box O3 (FOXO3; derived from its source gene) mediated by MDM2 (Du et al., 2017).

#### Template for translation

The idea that circRNAs can serve as templates for translation is based on their primary location in the cytoplasm (Li et al., 2018), which is supported by several studies (Legnini et al., 2017; Yang et al., 2017; Ho-Xuan et al., 2020; Li Y. et al., 2021). However, it was controversial because circRNAs were classified as noncoding RNAs previously. In fact, circRNAs perform this mechanism in two different manners, IRES (internal ribosome entry sites)-dependent and m^6^A-dependent (Yang et al., 2017), such as circ-ZNF609 (Legnini et al., 2017) and circARHGAP35 (Li Y. et al., 2021), respectively.

Among the three regulatory mechanisms mentioned above, miRNA sponging is the most frequently demonstrated action found in the inflammation of bovine mammary gland, which we will discuss in detail later. In addition, epigenetic modifications (N^1^-methyladenosine, 2′-O-ribosemethylation, inosine, 5-methylcytidine, N^6^-methyladenosine (m^6^A) and pseudouridine) in RNA molecules have captured researcher’s attention in recent years, and the functions and applications of these unique molecules have also been expanded upon (Wu et al., 2021; Xu et al., 2022). Following the deepening of circRNA research, the regulatory mechanism of ncRNA network in animal biological process will be clearer.

### The role of circRNAs in bovine mastitis

#### circRNA-miRNA-mRNA network

Research on circRNAs in mastitis has just begin, and their potential roles in regulating bovine mastitis are mainly based on a circRNA-miRNA-mRNA network model. It is reported that total 71 differentially expressed circRNAs are detected in LPS-stimulated MAC-T cells, and 2 significantly down-regulated circRNAs, novel_circ_000483 and novel_circ_0003097, are predicted to bind 19 miRNAs and 20 miRNAs, respectively, in immunological or inflammatory-related pathways, including the NOD-like receptor signaling pathway, bacterial invasion of epithelial cells, the MAPK signaling pathway, and the Notch signaling pathway (Wang J. P. et al., 2022). According to Chen et al. (2021), circRNA-08409 functions as a sponge of bta-miR-133a in cadmium-induced BMEC model, which targets TGFB2 (Recombinant Transforming Growth Factor Beta 2). Compared with the control, overexpression of circRNA-08409 significantly downregulated miR-133a and upregulated mRNA TGFB2 while inducing apoptosis. Subsequently, a NH_4_Cl-induced mastitis model was established, and the role of circRNA in regulating apoptosis and inflammation in bovine mammary epithelial cells was verified *via* the circ02771/miR-194b/TGIF1 axis (Chen et al., 2022). Both of the abovementioned studies not only illustrate that circRNAs can act as an indirect regulator in bovine mastitis by targeting complementary miRNAs, but also highlight that environmental chemicals could be potential factors for mammary gland inflammation in dairy bred management. Therefore, further efforts are required to investigate mastitis-related circRNAs and implement more innovative tools, like CRISPR-Cas13 based knock-down system (Li S. et al., 2021; Gao et al., 2022). This will undoubtedly speed up the progress of our research on understanding these unique molecules in mastitis.

#### Epigenetic modifications in circRNAs

In recent years, epigenetic modifications in circRNAs has become a new hotspot in RNA field, because its key biological function is to regulate circRNA metabolism, including biogenesis, translation, degradation, and cellular localization (Wu et al., 2021). However, there are very few studies on epigenetic modifications in mastitis-related circRNAs. In a recent study conducted by Xu et al. (2022), RNA immunoprecipitation and high-throughput sequencing (MeRIP-seq) were used for the first time to profile circRNAs regulated by m6A modification in mammary epithelial cells and the different pathological patterns between *S. aureus* and *E. coli* treatment were compared. Notably, the m^6^A-modified circRNAs, differentially expressed in the two groups were found to be highly similar in functional predictions, including immunity, cell junctions, growth metabolism, and resistance to bacterial invasion, indicating the compatibility and biomarker potency of circRNAs in mastitis.

## Emerging applications of noncoding RNAs in mastitis

The focus of this part is to summarize some of the emerging applications of noncoding RNAs (miRNAs and circRNAs) in disease prevention and treatments, and to look into their therapeutic potential in treating bovine mastitis.

### miRNA-based cross-species therapy

Most of the recent research focus on examining how changes in the abundance of a certain type or group of miRNAs inhibit or promote protein translation, while very few studies look at the impact of other molecular features on the various biological process, such as subcellular localization in cells (Jie et al., 2021) or organelles (Wang et al., 2020) and the differences in nucleotide lengths within the same isoform miRNA population. Notably, a recent study about the role of miRNA nucleotide length in cancer cell proliferation suggested that in addition to changes in single transcriptome abundance, changes in other biological characteristics also deserve to be noticed since they might make a difference in the regulation of pathological processes. In a study conducted by Qi et al. (2022), researchers compared the differences across the length of certain miRNAs associated with cell cycle in cancer cells under normal physiological conditions *via* single cell RNA-seq (scRNA-seq), and their observations suggest that the deficiency of miRNA function due to differences in length plays a crucial role in cancer cell development. Furthermore, the researchers compensated for the deficiency of length by introducing a protein from the plant immune system to suppress the cell-cycle of cancer cells. The protein is RNA-dependent RNA polymerase (RDR), which is absent in vertebrates (Cao et al., 2014). Notably, the effect of RDR on miRNAs is broad spectrum, which overcomes the limitations of single target of traditional cell cycle drugs. Additionally, they proposed that cross-species exogenous proteins could be used for molecular targeted therapy, and that these interesting miRNAs may also have other unknown properties that widely affect various regulations in organisms. In light of this, the rediscovery of these miRNAs will help us develop new strategies to treat bovine mastitis.

### circRNA vaccines

Traditional mastitis vaccine has been shown to have high rates of immunization failure or insufficient immune response. Various reasons may contribute to the failure of traditional vaccine, such as poor immunogenicity of the antigen (Rainard et al., 2022b), the lack of a suitable drug delivery system (Wang K. et al., 2022), the effect of immune inductive sites (Rainard et al., 2022a), and errors due to manual operation. In recent years, differing from previous vaccine strategies that were based on biological carriers such as engineered bacteria or adenovirus (Lasaro and Ertl, 2009), or specific antigens combined with immune adjuvants (Liu et al., 2021; Campra et al., 2022), a new vaccine development platform based on nucleic acid [DNA (Vogel and Sarver, 1995) or RNA (Geall et al., 2013)] has emerged. Nucleic acid vaccines have been introduced as the third generation vaccines with the advantage that they are more prominent in inducing humoral immunity or cellular immunity and can provide better protection compared to traditional vaccines (Wadman, 2021). The advantage of modern vaccines like DNA-based vaccines is that a small dose can bring about longer-lasting antigen stimulation. However, the immunization rate is dependent on a rigid cellular distribution, as only the DNA vaccine that reaches the nucleus can be successfully translated into the corresponding antigen (Kutzler and Weiner, 2008).

Recent years have witnessed the emergence of RNA-based vaccine technology as a new research hotspot. Contrary to DNA-based vaccine, it is not strictly dependent on cellular distribution but it does require administration at a higher dose. However, there are several caveats in of RNA based vaccines that need to be addressed, such as the requirement of specific modification (Ψ, Pseudouridine) to the nucleic acid sequence during synthesis (Kariko et al., 2008), and degradation caused by RNases from the environment. Hence, strict storage, transportation and delivery conditions are required (Zhang et al., 2020).

With the discovery of circRNA, its high stability resulting from its special structure and the ability to be translated into proteins with aids of specific elements or modifications, researchers quickly realized that this special molecule has the potential to serve as a more suitable delivery system of novel vaccines such as the RNA baesd vaccine (Liu and Chen, 2022). In a recent study, the strategy of engineering circRNA autocatalysis (Wesselhoeft et al., 2018) has been applied to fuse the spike protein sequence from SARS-CoV-2 and protein translation elements with the circRNA molecule to develop a novel circRNA-based RNA vaccine, and its strong protection effect against the virus has been confirmed in subsequent animal experiments (Qu et al., 2022).

### circRNA adjuvants

Another major challenge to developing an effective vaccine for mastitis is the complexity of host-pathogen interactions in the mammary gland. Not only does mammary gland play a pivotal role in microbial invasion and defense (Rainard et al., 2022a), but it also secrets and synthesizes fatty acids in milk (Lu Q. et al., 2021). Notably, a growing body of studies suggests that host metabolic processes may be related to its own immunity regulation (Buck et al., 2017), and this crosstalk often involves the normal or abnormal differentiation of cells during inflammation (Palsson-McDermott and O’Neill, 2020). Consequently, these target substances are regarded as a potential avenue for developing vaccine adjuvants during metabolic process. At the same time, noncoding RNAs intersect and overlap with substances mentioned above to play regulatory roles in cell metabolism and development. This suggests that circRNAs are capable of becoming new platforms for adjuvant development.

### circRNA medicines

RNA-based molecular targeted therapy involves both the interference of RNAs and the regulation of specific proteins (Yu and Tu, 2022). CircRNAs are also capable of being translated, which may allow researchers to obtain antibodies against specific pathogens through manipulating circRNA translation. Recently, it has been revealed that the interference of non-protective antibody may explain the failure of *S. aureus* vaccine, which suggests a delicate competition between antibodies targeting different subdomains of antigen (Tsai et al., 2022). This strategy has also been applied in a study discussed earlier (Qu et al., 2022), which undoubtedly enhances another possibility for utilizing the platform based on RNA-level treatment strategies.

## Conclusion and prospects

The struggle between pathogen and host has been a long-standing and important issue, especially since the eruption of coronavirus in 2019. This problem also persists in century-old animal husbandry. In recent years, with the continuous exploration of animal economic traits, the breeding business has improved rapidly. While productivity of animals has boosted, the cost of breeding and the difficulty of obtaining animal products have continued to decrease. Subsequently, sick animals are brutally eliminated and unqualified animal products are discarded as prevention and control measures for animal diseases.

Researchers have been increasingly focusing on the development of pathogen-based inspection and quarantine technologies. Despite being feasible in terms of reducing economic losses, these measures do not offer long-term solutions. As animal welfare has been raising its voice more and more over the last few years, it is also worth thinking about how to ensure a healthier life for these animals. Our efforts should also be directed toward researching the pathogenesis of environmentally-induced animal diseases and the development of vaccines and therapeutic drugs.

**Figure 4:**
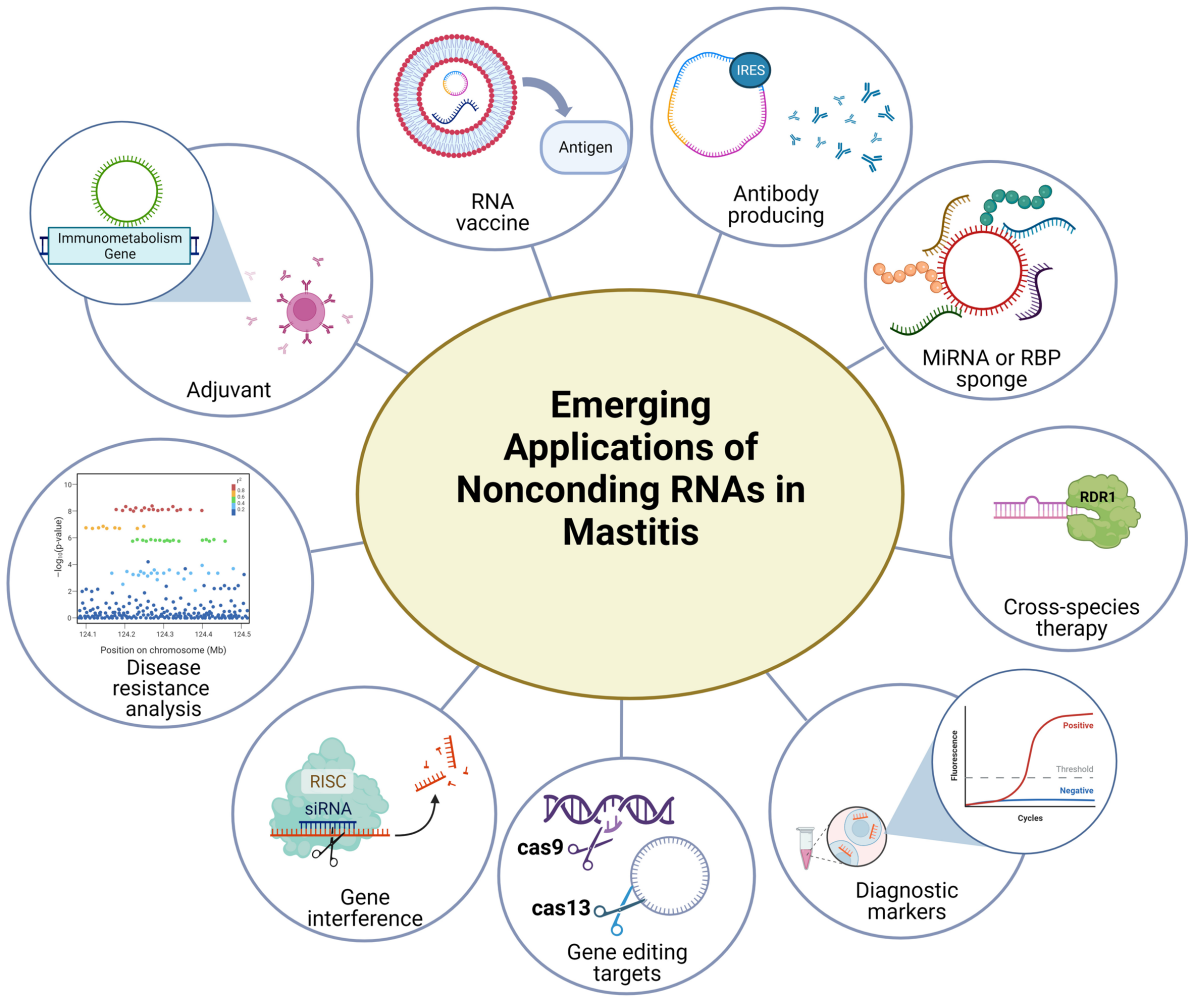
Emerging applications of noncoding RNAs in mastitis.

Dairy farming’s most common and costly disease is bovine mastitis, which has serious economic and animal welfare consequences. Exploring the regulatory mechanism behind bovine mastitis will no doubt benefit our understanding of this troublesome problem. Profiting from the progress in sequencing technologies, the breeding industry has come to a new era, and people have gained more insight on how to use new and key genetic selection markers to breed healthier and disease-resistant livestock. Noncoding RNAs as gene expression regulators have become a hot topic in research. Numerous co-working networks have been predicted but they are still in the theoretical stage since they have not yet been experimentally verified or functionally tested. Notably, new therapies based on noncoding RNAs are emerging and the development of delivery systems will widen the application of these novel platforms (Figure 4). There will be more breakthroughs in bovine mastitis if veterinary scholars and animal breeders collaboratively conduct comprehensive studies on noncoding RNAs in bovine mastitis.

## Author contributions

YY, ZY, and ZC contributed to conception and design of the study. WZ, TZ, BC, QL, XC, and KW collected and analyzed the data from previous studies. WZ and ZC wrote the first draft of the manuscript. YY revised the manuscript. All authors contributed to the article and approved the submitted version.

## Funding

This research was supported by Jiangsu Agricultural Science and Technology Independent Innovation Fund (CX(21)3119 to ZC), Education Department of Shaanxi Provincial Government (21JP110), Seed Industry Vitalization Program of Jiangsu Province (JBGS[2021]117 to YY), the “Qing Lan Project” (to ZC and YY) and the “High-end talent support program” (to YY) of Yangzhou University, China, and the Priority Academic Program Development of Jiangsu Higher Education Institutions (PAPD; to YY; for open access publication fees).

## Conflict of interest

The authors declare that the research was conducted in the absence of any commercial or financial relationships that could be construed as a potential conflict of interest.

## Publisher’s note

All claims expressed in this article are solely those of the authors and do not necessarily represent those of their affiliated organizations, or those of the publisher, the editors and the reviewers. Any product that may be evaluated in this article, or claim that may be made by its manufacturer, is not guaranteed or endorsed by the publisher.
